# Hand-Washing: The Main Strategy for Avoiding Hand, Foot and Mouth Disease

**DOI:** 10.3390/ijerph13060610

**Published:** 2016-06-18

**Authors:** Dingmei Zhang, Zhiyuan Li, Wangjian Zhang, Pi Guo, Zhanzhong Ma, Qian Chen, Shaokun Du, Jing Peng, Yu Deng, Yuantao Hao

**Affiliations:** 1 School of Public Health, Sun Yat-sen University, Guangzhou 510080, China; zhdingm@mail.sysu.edu.cn (D.Z.); lizhiyuan@mail.sysu.edu.cn (Z.L.); zhangwj35@mail2.sysu.edu.cn (W.Z.); guopi.01@163.com (P.G.); dengyu9@mail.sysu.edu.cn (Y.D.); 2 Department of Clinical Laboratory, Yuebei People’s Hospital, Shaoguan 512026, China; mazhanzhong816@163.com; 3 Department of Prevention and Health Care, Shijie Hospital of Dongguan, Dongguan 523290, China; chendu168@163.com (Q.C.); berrydudu@163.com (S.D.); 4 Baoan Center for Disease Control and Prevention of Shenzhen, Shenzhen 518101, China; pj077@163.com

**Keywords:** hand, foot and mouth disease, hand-washing, risk factors, case-control study, children

## Abstract

Epidemics of hand, foot and mouth disease (HFMD) among children have caused concern in China since 2007. We have conducted a retrospective study to investigate risk factors associated with HFMD. In this non-matching case-control study, 99 HFMD patients and 126 control from Guangdong Province were enlisted as participants. Data comprising demographic, socio-economic, clinical and behavior factors were collected from children’s parents through face-to-face interviews by trained interviewers using a standardized questionnaire. Results of the primary logistic regression analyses revealed that age, history of cold food consumption, hand-washing routines, and airing out bedding were significantly associated with HFMD cases. Results of further multivariate analysis indicated that older age (OR = 0.44, 95% CI: 0.34–0.56) and hand-washing before meals (OR = 0.3, 95% CI: 0.13–0.70) are protective factors, whereas airing out bedding more than thrice a month (OR = 4.55, 95% CI: 1.19–17.37) was associated with increased risk for HFMD. Therefore, hand-washing should be recommended to prevent HFMD, and the potential threat of airing out bedding should be carefully considered. However, further studies are needed to examine other possible risk factors.

## 1. Introduction

Hand, foot and mouth disease (HFMD) is a common acute infectious disease that is mainly caused by enteroviruses. In China, the major causative pathogens are *Enterovirus* 71 (EV71), *Coxsackievirus* A16 (CA16), and *Coxsackievirus* A6 (CA6) [1,2]. Transmission can occur through direct contact with nose and throat discharges, saliva, and blister fluid, as well as the stools of infected persons. Children under 10 years old, especially children younger than 5 years old, are the most susceptible population. The symptoms of HFMD are usually mild and self-limiting, but severe complications may occasionally occur, which can lead to fatal neurological, cardiovascular, and respiratory problems [3,4].

Outbreaks of HFMD occur worldwide, but these are more frequent in Asian countries [5]. In 2007, HFMD became a serious public health issue in China [6]. Later, in March 2008, a HFMD outbreak occurred in mainland China, resulting in a series of severe and fatal cases across multiple provinces, thereby raising serious health concerns nationwide. In response, the Chinese Ministry of Health designated HFMD as a nationally notifiable disease and placed it under standard management. A total of 13.83 million cases have been officially reported in mainland China from 2008 to December 2015 [7]. Equally striking, Guangdong, a southern province with a typical subtropical climate, is an ideal breeding ground for enteroviruses and accounted for 12.75% of all reported HFMD cases [8]. 

Until now, no effective chemoprophylaxis is available for HFMD. A vaccine for EV 71 has been recently developed, but has not been widely utilized [9]. Moreover, there is no multivalent vaccine against EV71, Cox A16, Cox A6, and other enterovirus types [10]. Public health prevention measures are still the most practical and effective means of reducing transmission [11,12]. In this circumstance, prevention focuses on underlying risk factors, and public health intervention continues to play an imperative role. Some studies have focused on certain aspects of HFMD risk factors. Ruan *et al.* [13] mainly focus on behavior factors and concluded that hand-washing had a significant protective effect, while Lin *et al.* [14] indicated that exclusive breast feeding is a protective factor against the infection. Xie *et al.* [15] analyzed the importance of public playgrounds in the transmission of HFMD, and Deng *et al.* [8] explored environmental risk factors of HFMD. However, most of these studies only focus on some expects of potential risk factors of HFMD. Behavior factors including cold food consumption, habits of using pacifier and airing out bedding, and clinical factors like birth conditions were not mentioned in previous studies. Therefore we conducted this study to analyze the HFMD cases in Guangdong, to fully examine the risk factors of HFMD. The study finding may have implications for further research and public health intervention.

## 2. Materials and Methods 

### 2.1. Study Design and Settings

This study is a non-matching case-control study focusing on HFMD cases among children 10 years old and younger. Cases were selected from Shijie Hospital of Dongguan City and Yue Bei People’s Hospital in Guangdong Province in between January 2015 and December 2015. 

We used the case definition of HFMD given in the “Guideline for the diagnosis and treatment of hand foot and mouth disease (2010 ed.)” issued by the Chinese Ministry of Health [16]. HFMD is defined as a condition that “may or may not show fever; is accompanied by vesicular or papular rashes on the hands, feet, and buttocks; and is confirmed by laboratory enteroviruses (EV71, CA16, or other enteroviruses) or neutralizing antibody tests.” Stratified random samples of control data were selected from the same hospital or the same kindergarten for inclusion. Medical examination notification and informed written consent was obtained before each interview. And the study was approved by the Ethics Committee of the School of Public Health, Sun Yat-sen University (Project Identification Code: (L2016) 021).

### 2.2. Questionnaire and Interview

Standard questionnaire was used for data collection in face-to-face interviews administered by trained interviewers. Interviews were administered to the guardians of the subjects. The collected data comprised demographic factors (sex, age), socio-economic factors (education , whether the child went to school/kindergarten or not, and whether the child was a permanent resident or part of the floating population, whether there is any other children in the family, who is the caregiver), clinical factors (birth and feeding conditions including birth weight, recent vaccination history, recent history of common cold and diarrhea, and history of HFMD), and behavior factors (history of cold food consumption, whether he/she has been exposed to populated places, sharing toys with other children, history of contact with common cold or diarrhea patients, hand-washing habits, medical-seeking conditions, habits of airing out bedding, whether there is a dedicated towel for the child, and pacifier-using and finger-sucking conditions). All sets of information on risk factors were truncated to the date of onset for cases and to the date of the interview for the control subjects. Medical files were reviewed as needed for clarification. Considering the incubation period of HFMD, we probed the most exposures during a two week period leading to the onset of the disease for the cases or before the interview for the control subjects.

### 2.3. Statistical Analysis

Database was built by EpiData3.1 (The EpiData Association, Odense, Denmark), and statistical analysis was performed by SPSS19.0 (SPSS Inc., Chicago, IL, USA). Descriptive analysis was performed for demographic information. Differences among the means of continuous variables were tested using Student’s *t*-test if the sets of data were normally distributed. Otherwise, the Kruskal-Wallis method was used. Univariate logistic regression analysis was first performed to examine the odds ratios (OR) for risk factors. Then age was included in the logistic regression analysis to adjust confounder effect. A further multivariate logistic regression analysis was then performed to examine the adjusted odds ratios for risk factors that were significant in previous multivariate analyses. A *p* value of <0.05 was considered statistically significant. Moreover, the receiver operating characteristic (ROC) curve was fitted to the observed data, and the accuracy of the model was measured by the area under the curve (AUC) [17].

## 3. Results

### 3.1. Participant Characteristics

Characteristics of both case and control participants were summarized in Table 1. During the study period, 99 cases and 129 control subjects were recruited. A total of 228 questionnaires were sent and 227 responses were collected, among which, 222 had complete information (97.4%), including 96 for the case group and 126 for the control group. The final response rates for cases and controls were 96.97% and 97.67%, respectively. There is no difference between the response rate among the two groups (*p* = 1). Overall, 126 male participants and 96 female participants were enrolled. Gender distributions were similar between the two groups. The average age (±SD) of participants is 4.47 (±2.07) years old, and participants infected with HFMD were younger than those in the control group. The age of cases were ranged from 1 to 8 years old, while the age of controls were ranged from 0 to 10 years old.

### 3.2. Risk Factor Analysis

The results of univariate logistic regression are summarized in Table 2. In the unadjusted univariate analysis, the cases and controls were significantly different in the following 11 aspects: education, whether the child is a permanent resident or part of the floating population, recent vaccination history, history of HFMD, history of cold food consumption, exposure to populated places, history of contact with common cold or diarrhea patients, hand-washing before meals, hand-washing after toilet use, pacifier-using, and finger-sucking conditions. Considering the effects of age, we further found that case and control groups were significantly different in the following three areas: history of cold food consumption (OR = 0.42, 95% confidence interval (CI): 0.20–0.88), hand-washing before meals (OR = 0.41, 95% CI: 0.19–0.89), and airing of bedding more than thrice per month (OR = 3.83, 95% CI: 1.08–13.58).

**Figure 1:**
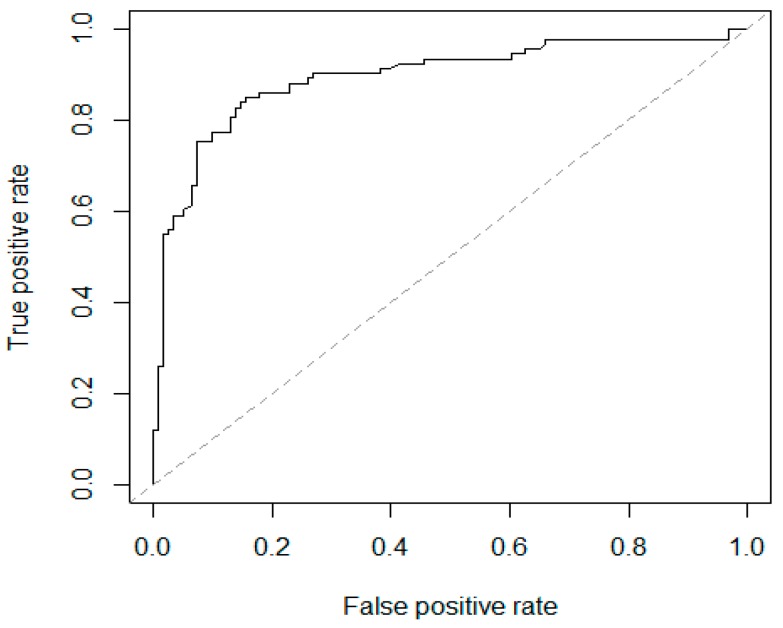
The receiver operating characteristic curve of age, hand-washing before meals and airing out bedding for the ability to differentiate the HFMD cases from the control individuals. AUC: 0.895.

A multivariate logistic regression was performed to further investigate the association between HFMD and the four factors identified by the previous tests. Results are summarized in Table 3. The multivariate analysis identified the following three factors: age, hand-washing before meals, and airing out bedding. Among these factors, hand-washing before meals was associated with a significantly lower risk of HFMD infection (OR = 0.30, 95% CI: 0.13–0.70). However, airing of bedding was related to a higher risk of HFMD. The risk of getting HFMD is 4.55, 3.23, 3.10, and 1.84 times higher for children whose families aired bedding more than thrice per month, twice per month, and once per month, respectively, when compared with children whose families aired bedding less than once per month. The ROC curve of the modified logistic model is demonstrated in Figure 1, with an AUC of 0.895.

## 4. Discussion

HFMD caused by enteroviruses continues to be a threat among Asian children [18]. As no universal vaccine is available for enteroviruses and there is a lack of effective chemoprophylactic treatment against HFMD, identification and prevention of underlying risk factors is still the key to reduce its transmission. Our findings suggested that hand-washing before meals is related with a significant reduction of HFDM risk. This protective association of hand-washing and HFMD is not surprising. Enteroviruses are transmitted predominantly via the fecal-oral route, and contacts with contaminated saliva, vesicular fluids, and fomites, while contaminated hands play an important role in this process [19]. In addition, enteroviruses achieve optimum growth at 37 °C and are resistant to acidic pH and detergents. Therefore, it is possible for the viruses to survive in the hands for a relatively long period of time [18]. These findings are also consistent with those obtained in previous studies conducted by Ruan *et al.* [13] and Xie *et al.* [15], both of which supported the importance of hand-hygiene in preventing HFMD. Moreover, a dose-response effect between hand-washing and a lower risk of HFMD infection was reported [13]. Hand-washing is also an effective intervention in preventing other infectious diseases. Its protective effects ranged from 34% for impetigo to 53% for diarrhea according to a randomized controlled trial [20]. Our study further emphasized the importance of hand-washing and reported that it is related with a 70% risk reduction for HFMD.

Furthermore, we found that airing out bedding is associated with an increased risk for HFMD and a dose-response effect was also observed. Airing out bedding under the sun is very common among Chinese residents to lower the humidity and dust mite levels of bedding. It is also recommended by some local Centers for Disease Control and Prevention as an intervention to prevent HFMD [21]. However, we discovered airing out bedding more than thrice per month significantly increases the risk of HFMD by 4.55 times, compared with a frequency of less than once per month. Therefore, it should be noticed that airing out bedding could be a potential risk factor of HFMD. The association between airing out bedding and risk of HFMD can be explained by the persistent characteristics in the environment of enteroviruses and their transmission patterns [19,22,23]. Although the major infectious source of HFMD was patients, touching virus-carrying bedclothes can also lead to infections. We speculated that airing out bedding may increase the chance of exposing bedding to fomites in the external environment, and fomites may also spread from one to other bedclothes during this process. Therefore, we recommended that bedding should be put in a clean and uncrowded place while airing. Furthermore, washing bedding with disinfectants containing oxidants and drying them thoroughly in a clean environment may be a better option [24]. Besides, we should not ignore the possibility of potential confounder effects. It is possible that families with low socio-economic status are living in places with poor hygiene. Therefore, when the bedding is put outside, there is a higher chance to get contaminated. In addition, we also noticed that crude OR and adjusted OR of airing out bedding were quite different. We performed a Spearman’s Rank Order Correlation to investigate the correlation between airing out bedding and age. The Spearman’s correlation coefficient is −0.329 and the result is significant (*p* value < 0.001). The results showed that the family has younger child tended to airing out their bedding more often. 

We also discovered that a recent history of cold food consumption may be related to protective effects, which were seldom mentioned in the previous studies. The cold food in our study refers to cold dish, sushi and ice-cream but not including fruits. This finding is inconsistent with a few current prevention guidelines in China [25]. Since enteroviruses can survive long in low temperatures and can be inactivated by heat treatment, it may be safer to consume cooked food rather than cold food. However, casual relationship between cold food consumption and HFMD cannot be confirmed by the study results since it is a cross-sectional study. In this correlation, children who are healthier may have better immunity and have lower chances of getting infections from eating cold food, while less healthy children may not be given cold food by caregivers [26]. For this factor, we suggested that the key is proper food handling, regardless if the food is cold or cooked. On the other hand, it is possible that children have more chance to eat cold food may have higher socio-economic status. History of cold food consumption may be a mediator, while socio-economic status may be the main effect in this correlation.

Our findings have both academic value and practical significance. These results provide epidemiological evidence of the benefits of hand-washing and support the importance of hand-washing to prevent HFMD and other infectious diseases. This study also recognized the potential harm of airing out bedding, which was not mentioned in previous studies. In addition, with an AUC of 0.895, the final logistic model showed a good capacity of separating children with high risk of HFMD infection from the control group. For better conclusion, this study covered a more comprehensive range of possible risk factors, namely, demographic factors, birth and feeding conditions, and living habits of child and his/her caregivers. 

A few limitations of this study need to be considered. We selected the control subjects from the hospital information system and kindergartens randomly, instead of performing a matching case-control study, which may have led to a potential selection bias. To solve the problem of age distribution being different among the two groups, we adjusted the age in the analysis to eliminate the possible confounding effects. The study is also suffered from Berkson’s bias. The ORs in our results may be lower than the real value since some of the controls are unhealthy children with other diseases. These children may have poor immunity and high risk to get HFMD. Moreover, only HFMD patients who went to hospital were included as cases, which may not be representative for all HFMD infections. Recall bias should not be a serious problem in our study, as most exposures were determined retrospectively from the recall of caregivers. Interviews for the cases were performed when they went to the doctor, and the questions only covered the occurrences two weeks prior to the interview, which may have reduced the recall bias. Besides, as a cross-sectional study, we don’t have sufficient evidence to establish a causal relationship between risk factors and HFMD infection. Further randomized control trial or cohort study may be needed to provide stronger evidence. Another limitation is that we did not have a more detailed set of information describing the methods of hand-washing. The difference between the protective effects of hand-washing using water only and hand-washing with soap should be further explored. Socio-economic factors were not included as a risk factor, which is closely related with personal and environment hygiene. But we investigate whether being a permanent resident have any effect on HMFD infection, which may reflect the participant’s socio-economic status in some extent. 

## 5. Conclusions

This study suggests that hand-washing is a main strategy to prevent HFMD infection. Moreover, we need to be more careful when recommending airing out bedding as to the public. Further studies are warranted in the future because of potential limitations.

## Figures and Tables

**Table 1:** Demographic characteristics of the study participants.

Characteristics	Cases (*n* = 96)	Controls (*n* = 126)	*p* Value
Male	34	58	0.88
Age, years (mean ± SD)	2.92 ± 1.68	5.59 ± 1.53	<0.01

SD: standard deviation.

**Table 2:** Univariate and multivariate analyses on factors associated with HFMD infection in preschool-aged children adjusted by age.

Risk Factor		Case (*n* = 96)	Control (*n* = 126)	OR	95% CI	Adjusted OR	Adjusted 95% CI
**Socio-economic factor**							
Education	School children	3	9	0.81	0.02–0.34	3.31	0.43–25.46
	Preschool children	30	101	0.72	0.04–0.14	0.85	0.27–2.66
	Children raised at home	62	15	1	-	1	-
Whether the child is permanent resident or floating population	Permanent resident	78	120	0.72	0.02–0.32	0.18	0.03–1.04
	Floating population **^a^**	18	2	1	-	1	-
Any other children in the family	Yes	49	64	0.98	0.57–1.67	1.17	0.58-2.36
	No	47	60	1	-	1	-
Who is the caregiver	Father	6	4	3	0.20–45.24	20.50	0.82–514.48
	Mother	51	75	1.36	0.12–15.40	3.49	0.22–55.47
	Grandparents	38	43	1.77	0.15–20.27	5.03	0.31–82.46
	Baby-sitter	1	2	1	-	1	-
**Clinical factor**							
Birth weight	-	-	-	0.80	0.51–1.23	0.61	0.34–1.09
Recent vaccination history	Yes	41	18	4.33	2.26–8.30	1.58	0.63–3.95
	No	51	97	1	-	1	-
Recent history of common cold and diarrhea	Yes	37	46	1.06	0.61–1.84	0.91	0.5–1.88
	No	59	78	1	-	1	-
History of HFMD	Yes	6	22	0.31	0.12–0.80	0.33	0.11–1.03
	No	-	-	1	-	1	-
**Behavior factor**							
History of Cold food consumption **^b^**	Yes	24	68	0.28	0.16–0.51	0.42	0.20–0.88
	No	69	55	1	-	1	-
Whether he/she has been exposed to populated places	Yes	58	57	1.87	1.09–3.22	1.35	0.67–2.74
	No	37	68	1	-	1	-
Sharing toys with other children	Yes	73	91	1.24	0.67–2.30	1.79	0.77–4.17
	No	22	34	1	-	1	-
History of contact with common cold or diarrhea patients	Yes	29	20	2.9	1.49–5.67	2.10	0.87–5.09
	No	47	94	1	-	1	-
Medical-seeking conditions	Yes	31	35	1.28	0.72–2.29	1.27	0.59–2.72
	No	63	91	1	-	1	-
Recent history of hospital visit (Family members)	Yes	35	39	1.28	0.73–2.24	1.45	0.68–3.08
	No	61	87	1	-	1	-
Hand-washing before meals	Often	50	102	0.54	0.01–0.44	0.41	0.19–0.89
	Seldom or not	46	24	1	-	1	-
Hand-washing after toilet use	Often	60	102	0.39	0.21–0.72	0.79	0.35–1.75
	Seldom or not	36	24	1	-	1	-
Habits of airing out bedding	More than thrice	27	9	3.18	1.16–8.67	3.83	1.08–13.58
	Thrice	12	14	0.91	0.33–2.51	2.38	0.59–9.63
	Twice	16	19	0.90	0.35–2.28	2.90	0.78–10.76
	Once	24	66	0.39	0.17–0.87	1.37	0.45–4.21
	Less than once	17	18	1	-	1	-
Whether there is a dedicated towel for the child	Yes	90	124	0.24	0.05–1.23	0.68	0.10–4.79
	No	6	2	1	-	1	-
Pacifier-using	Yes	49	23	4.62	2.53–8.46	0.78	0.32–1.89
	No	47	102	1	-	1	-
Finger-sucking conditions	Often	30	7	10.91	4.36–27.26	3.02	0.89–10.30
	Occasionally	33	34	2.47	1.32–4.62	1.24	0.56–2.73
	No	33	84	1	-	1	-

**^a^** Floating population refers to migrants without local household registration status (hukou); **^b^** Cold food refers to cold dish, sushi and ice-cream, not including fruits.

**Table 3:** Further multivariate analysis on factors associated with HFMD infection in preschool-aged children.

Risk Factor		*p* Value	OR	95% CI
Age	-	0.00	0.44	0.34–0.56
History of cold food consumption	Yes	0.06	0.47	0.22–1.02
	No	-	1	-
Hand-washing before meals	Often	0.01	0.30	0.13–0.70
	Seldom or not	-	1	-
Habits of airing out bedding	More than thrice	0.03	4.55	1.19–17.37
	Thrice	0.14	3.23	0.68–15.31
	Twice	0.11	3.10	0.78–12.34
	Once	0.31	1.84	0.56–6.04
	Less than once	-	1	-

## Abbreviations

The following abbreviations are used in this manuscript:

HFMD	hand, foot and mouth disease
EV71	enterovirus 71
CA16	coxsackievirus A16
CA6	coxsackievirus A6
OR	odds ratio
ROC	receiver operating characteristic
AUC	area under the curve
CI	confidence interval
